# Oral Glutamine May Have No Clinical Benefits to Prevent Radiation-Induced Oral Mucositis in Adult Patients With Head and Neck Cancer: A Meta-Analysis of Randomized Controlled Trials

**DOI:** 10.3389/fnut.2020.00049

**Published:** 2020-04-17

**Authors:** Ting Shuai, Xu Tian, Ling-Li Xu, Wei-Qing Chen, Yuan-Ping Pi, Lin Zhang, Qiao-Qin Wan, Xiu-E Li

**Affiliations:** ^1^Second Dental Center, Peking University School and Hospital of Stomatology, Beijing, China; ^2^Department of Gastroenterology, Chongqing University Cancer Hospital, Chongqing, China; ^3^Department of Nursing, Peking University School and Hospital of Stomatology, Beijing, China; ^4^School of Nursing, Peking University, Beijing, China

**Keywords:** oral mucositis, head and neck cancer, radiotherapy, glutamine, systematic review

## Abstract

**Objectives:** The role of oral glutamine for the management of oral mucositis (OM) has not yet been confirmed. The objective of the present study is to further investigate whether oral glutamine is effective in preventing and treating OM among patients with head and neck cancer (HNC) receiving radiotherapy alone or concurrent with chemotherapy.

**Methods:** A systematic search was performed in PubMed, EMBASE, EBSCO, and Cochrane Central Register of Controlled Trials (CENTRAL) to capture all potential citations from the inception to June 2019. Then data extraction and assessment of risk of bias were carried out after selecting the eligible citations. RevMan 5.3 software was used to perform all statistical analyses.

**Results:** Six randomized controlled trials (RCTs) including 441 patients were included in the final analysis. The meta-analysis showed that oral glutamine couldn't significantly decrease the incidence of OM (risk ratio [RR] = 0.98, 95% confidence interval [CI] = 0.94−1.02) and alleviate the development of moderate or severe grade of OM (Moderate-to-severe OM: RR = 0.81, 95% CI = 0.59−1.12; Severe OM: RR = 0.45, 95% CI = 0.13−1.52). But oral glutamine may have the potential to reduce the opioid use (RR = 0.84, 95% CI = 0.71−0.99). The role of oral glutamine in delaying the onset of OM remains uncertain due to conflicting results between quantitative (mean difference [MD] = 4.11 days, 95% CI = 3.49−4.73) and qualitative results.

**Conclusions:** Oral glutamine may have no clinical benefits to prevent or reduce the incidence and severity of radiation-induced OM in patients with HNC receiving radiotherapy alone or concurrent with chemotherapy. It is also uncertain whether oral glutamine can delay the onset of OM. But it may have the potential to relieve the degree of oral pain. Nevertheless, we must cautiously interpret the results because the observed effect size for delay in mucositis start or reduction in opioid use is marginal. Moreover, further RCTs with more rigorous methodology and large-scale are required to enhance the quality of evidence.

## Introduction

Issued data showed that head and neck cancer (HNC) was ranked at eighth among all cancers and the new cases were more than 710 thousand in 2018 (1). Radiotherapy (RT) is one of the three cornerstones in the treatment of HNC (2). However, RT regime usually damage the integrity of the normal tissue (3). Oral mucositis (OM) is viewed as the most prevalent and troubling side effect in HNC patients receiving RT, which results from the direct toxic damage of RT with or without chemotherapy (CT) on oral mucosa (4). Approximately 80% of HNC patients will experience OM during RT, which is much higher than that in most cancers such as esophageal cancer (57.8%) and colorectal cancer (63%) (5, 6). Especially when cumulative doses of RT are larger than 30 Gy in the field of oral mucosal and patients receive CT simultaneously, nearly 100% of the HNC patients may develop OM (7).

During the treatment of HNC, patients' quality of life (QoL) is the fundamental concern. However, OM will cause a series of uncomfortable problems including pain, troubles in eating and swallowing, malnutrition, and speech problem, which significantly reduces QoL (8, 9). Moreover, moderate or severe OM may delay RT regime or limit the doses of RT regime, which may worse the prognosis of HNC patients (10, 11). Hence, this is the primary goal to prevent and alleviate OM as much as possible for HNC patients receiving RT alone or with CT.

At present, there are plenty of published studies exploring different ways for the prevention of OM, such as laser therapy or several nonpharmacologic methods (12–14). But there is still lack of definitive conclusion about the effect of these interventions. Therefore, some researchers are still devoted to develop the appropriate ways for the prevention and alleviation of OM. As the most abundant free amino acid, glutamine plays a critical role in providing precursor nitrogen to synthesize purines and pyrimidines (15). And so glutamine is an important material to maintain the metabolic homeostasis during stress (16). Evidence suggests that glutamine may have an effect on the preservation of mucosal structure after RT damage and speculated to be beneficial for RT-induced OM (17, 18).

To date, there are several randomized controlled trials (RCTs) evaluating the effect of oral glutamine on preventing RT-induced OM in HNC patients. Among these trials, four studies demonstrated oral glutamine was effective for decreasing the onset and the severity of OM in HNC patients (19–22). But Huang et al. and Lopez-Vaquero et al. find no beneficial effects of oral glutamine in decreasing the incidence, onset and severity of OM (23, 24). Although four published systematic reviews (25–28) explored the impact of glutamine use on OM induced by cancer treatment, the definitive conclusion has not yet been generated due to several limitations. More importantly, additional eligible studies have been recently published (20, 23, 24). So it is necessary to perform an updated systematic review and meta-analysis to combine all evidence to further investigate the role of oral glutamine in preventing RT-induced OM in HNC patients receiving RT alone or concurrent with CT.

## Methods

The present systematic review and meta-analysis was carried out according to the methods recommended by the Cochrane Collaboration (CC) (29). We reported all statistical results in accordance with the framework published in the Preferred Reporting Items for Systematic Reviews and Meta-Analysis (PRISMA) statement (30). And it has been registered in the International Prospective Register of Systematic Reviews (PROSPERO) platform with an unique number of CRD42019139093 (31).

### Literature Retrieval

In order to search all relevant literatures, electronic, and manual searching were combined. On the one hand, four databases including PubMed, EMBASE, Cochrane Center Register of Controlled Trials (CENTRAL) and EBSCO were retrieved from inception to June 2019. On the other hand, the reference lists of all eligible studies and related reviews were also manually checked to obtain additional eligible studies. The search algorithms were constructed using the combination of Medical Subject Headings (MeSH) and text words. The key words included “glutamine,” “oral mucositis,” and “random^*^.” Two investigators (TS and LLX) were independently assigned to search literature in four databases. Search algorithms were documented in Supplemental Table 1. Endnote X7.0 software was applied to managing all literature. The last search was updated in August 2019.

### Inclusion Criteria

We designed inclusion criteria as follows according to patients, intervention, comparison, outcomes, and study design (PICOS): (a) All adults with biopsy-proven HNC such as oral cancer or nasopharyngeal cancer, who received RT with or without CT; (b) Oral glutamine regardless of dose was offered in the treatment group and placebo or nothing was provided in the control group; (c) The primary outcomes were the incidence and severity of OM and the secondary outcomes were the onset of OM, oral pain and glutamine-related adverse events; (d) Only RCTs were eligible in the present study. Abstracts with enough data could also be included. We only included studies published in English.

### Exclusion Criteria

We excluded a study if it met at least one of the following criteria: (a) Patients had history of RT or CT, or had developed OM; (b) Patients were instructed to intravenously admitted glutamine (32) or firstly swish and then expectorate glutamine (33); and (c) The duplication without sufficient information and poor quality or animal experiment.

### Definition of Outcomes

The incidence of OM was set as the number of all OM cases in each group. The severity of OM was evaluated by the grade of OM which was scored according to Common Terminology Criteria Adverse Events (CTCAE) or World Health Organization (WHO) method. Grade 2 was considered as moderate and grade 3 or 4 severe (27, 34). Meanwhile, the onset of OM was defined as the time at which the patient was diagnosed with OM. Oral pain was assessed by the incidence of opioid use and pain scores. Glutamine-related adverse events were reported by individual study.

### Data Extraction

Two investigators (XT and LZ) were independently assigned to review title and abstract of each study in order to judge the eligibility according to selection criteria. If a study initially met inclusion criteria, full-text was accessed to further check its eligibility. The same two investigators extracted all essential information with the predesigned data extraction sheet (Microsoft™). The extraction information included the leading author, country, publication year, type of cancer, age and sex of patients, details of RT regime, intervention regimes, OM scoring system and outcomes of interest. If there was any disagreements, Xiu-E Li would make the final decision.

### Risk of Bias of Included Studies

Two independent investigators (TS and QQW) used the Cochrane risk of bias assessment tool to appraise the risk of bias of each study from the seven aspects as following: randomization sequence generation, allocation concealment, blinding of participants and study personnel, blinding of outcome assessors, incomplete outcome data, selective reporting, and other biases (35). Finally, we graded the overall quality of each eligible study as moderate if most of the eligible studies were labeled as unclear or low risk of bias. Xiu-E Li would solve the discrepancy between the two investigators.

### Statistical Analysis

Mean difference (MD) with 95% confidence interval (CI) was used to express the continuous data, and relative risk (RR) with 95% CI was used to calculate the categorical data. In the present study, we used random-effect model, which simultaneously combine the heterogeneity of within and across trials to perform all statistical analyses (36). The heterogeneity was qualitatively described by chi-square test, and quantitatively estimated by I^2^ statistic which can estimate the proportion of the overall variation that is attributable to across study heterogeneity (37, 38). When the number of included studies for individual outcome was less than 10, the funnel plot was not drawn (39). Two independent investigators (QQW and TS) used RevMan5.3 software (Copenhagen, Denmark: The Nordic Cochrane Centre, The Cochrane Collaboration) to perform all analysis.

## Results

### Results of Study Retrieval and Selection

**Figure 1:**
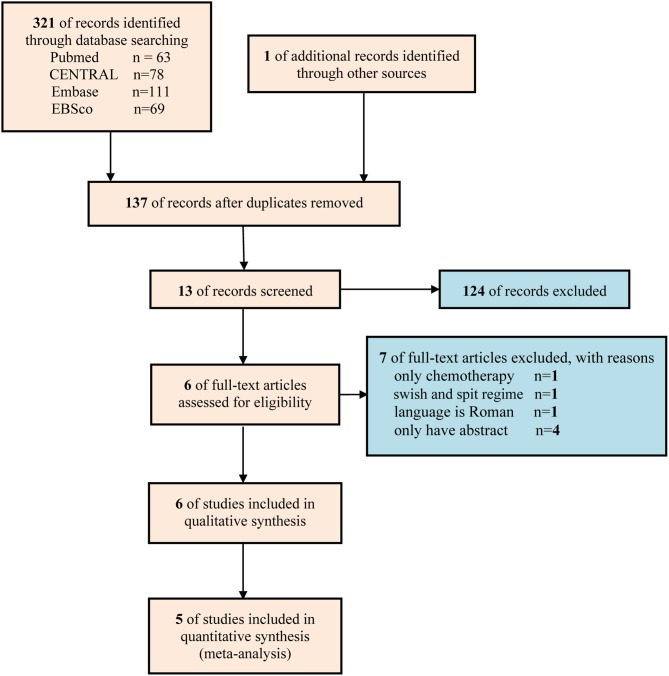
Flow chart of study retrieval and selection.

A total of 322 studies were captured at the first literature retrieval stage. Finally only 6 studies (19–24) involving 441 patients were eligible for qualitative analysis, and 5 studies involving 279 patients were included in meta-analysis (19, 20, 22–24). The flow diagram of retrieval and selection of literature was delineated in Figure 1.

### The Characteristics of Included Studies

The basic characteristics of the 6 studies were summarized in Table 1. For the six studies included in qualitative analysis, the sample size of individual study ranged from 20 to 81 and the RT doses were ranging from 60 to 70 Gy. The doses and frequency of oral glutamine were slightly different. Moreover, four studies (20, 22–24) used CTCAE to evaluate the grade of OM and only one study (19) used WHO method, the other one (21) did not reported the method of evaluating OM. All studies reported that the baseline information between the glutamine group and the control group was not statistically significant.

**Table 1:** The basic characteristics of the six included studies.

**References**	**Country**	**Age (years)**		**Sample size** **(M/F)**		**Details of RT regime**	**Detailed summary of intervention regimes**		**Scoring system**	**Outcomes**
		**TG**	**CG**	**TG**	**CG**		**TG**	**CG**		
Huang et al. (23)	China	52.2 ± 9.5	52.6 ± 10.3	28/3	32/1	RT of 60–66 or 70 Gy in 2 Gy fractions, once per day, 5 day/week.	10 g L-glutamine and 5 g maltodextrin dissolved in cold water 30 min before a meal, 3 times/day, beginning 1 wk before RT, during RT, and for 2 wks after completion of RT.	15 g maltodextrin	CTCAE	Incidence and severity of OM;
Diwan and Khan (20)	India	47 ± 8.5	52 ± 10.5	19/11	16/14	RT of 66 to 70 Gy in 1.8 to 2 Gray fractions, once per day, 5 fractions/week.	10 g glutamine, dissolved in 2 glasses of water, twice/day (within 1 h before radiation and 7 to 8 h post radiation), 5 days/wk and only on RT days.	Placebo	CTCAE	Incidence of opioid use
Lopez-Vaquero et al. (24)	Spain	61.5 (32-81)	59 (39-78)	18/7	20/5	70 Gy in 35 fractions of 2 Gy, or 66 Gy in 30–33 fractions of 2 Gy.	10 g L-glutamine three times/day, dissolved in a glass of water.	10 g maltodextrin	CTCAE	Incidence and severity of OM;
Pattanayak et al. (21)	India	52.2 ± 7.3	53.5 ± 6.9	54/27	59/22	RT of 66 Gy in 33 fractions or 70 Gy in 35 fractions on Monday through Friday over 7 weeks.	15 g glutamine in a glass of water, twice/day throughout treatment.	Negative or control subjects	Unclear	Incidence of opioid use
Tsujimoto et al. (22)	Japan	60.5 ± 10.8	63.2 ± 5.4	17/3	17/3	RT of 60 or 70 Gy in 2 Gy fractions, once per day, 5 fractions/week.	10 g glutamine 3 times/day (at 7:00, 11:00, and 16:00 h) throughout the RT course.	Placebo	CTCAE	Incidence and severity of OM
Chattopadhyay et al. (19)	India	56 ± 12.2	57.8 ± 14.6	26/9	24/11	Total radiation dose was not reported. RT in 2 Gy fractions, once per day, 5 days weekly.	10 g glutamine dissolved in 1 L of water, within 2 h before radiation, once a day, 5 days/wk on treatment days.	Nothing	WHO	Incidence and severity of OM

*TG, treatment group; CG, control group; M, male; F, female; RT, radiation therapy; OM, oral mucositis; CTCAE, Common Terminology Criteria Adverse Events; WHO, world health organization*.

### Quality Assessment of Included Studies

**Figure 2:**
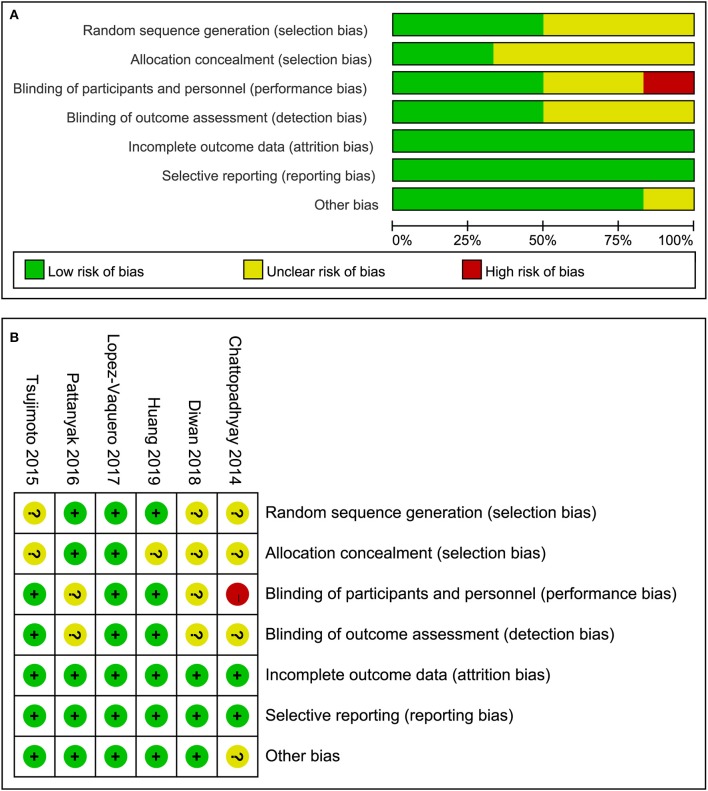
Risk of bias. **(A)** risk of bias graph and **(B)** risk of bias summary.

All the included studies reported randomization but only three studies (21, 23, 24) adequately introduced the method of generating randomization sequence. Only two studies (21, 24) reported the methods of allocation concealment. Of these six studies, Chattopadhyay et al. (19) set blank control regime and it was impossible to perform the blinding of participants and study personnel. Therefore the domain of the study was considered as a high risk of bias. Moreover, Chattopadhyay et al. (19) designed a randomized and prospective single institutional case control study and it may be a potential source of other bias. But we couldn't contact the lead author to confirm the bias so we defined the domain of other bias as unclear risk of bias. In summary, the overall methodological quality of all included studies was considered as moderate level. The evaluation results of risk of bias were depicted in Figure 2.

### Incidence of OM

**Figure 3:**
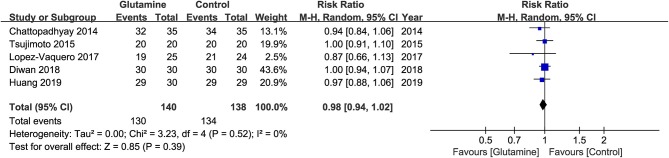
Meta-analysis of incidence of total OM.

The five eligible studies (19, 20, 22–24) involving 279 patients reported the incidence of OM at the six week of RT. The meta-analysis suggested that the incidence of OM was not statistically significant between the two groups (RR, 0.98; 95% CIs, 0.94 - 1.02; *P* = 0.39; I^2^ = 0%; seen as Figure 3).

### Severity of OM

**Figure 4:**
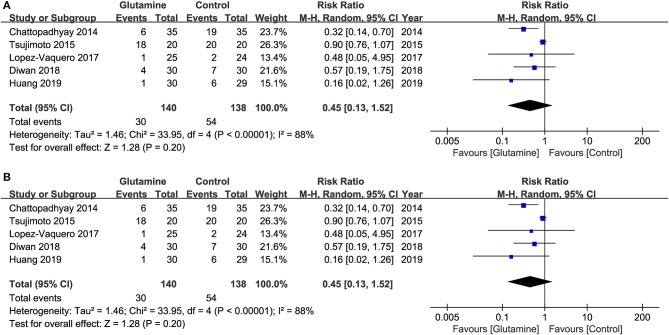
Meta-analysis of incidence of various OM. **(A)** the incidence of moderate and severe OM and **(B)** the incidence of severe OM.

Five studies (19, 20, 22–24) reported a moderate and severe grade of OM, which revealed the severity of OM. The results showed that the incidence of moderate and severe OM, and the incidence of severe OM were both not statistically significant between the two groups (Moderate and severe OM: RR, 0.81; 95% CIs, 0.59–1.12; *P* = 0.20; I^2^ = 88%, seen as Figure 4A; Severe OM: RR, 0.45; 95% CIs, 0.13–1.52; *P* = 0.20; I^2^ = 88%, seen as Figure 4B). Moreover, Pattanyak et al. (21) also reported the severity of OM. In this study, specific data about this outcome was reported, but authors qualitatively described that oral glutamine was effective on decreasing the severity of OM.

### Onset of OM

**Figure 5:**
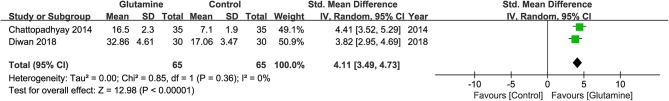
Meta-analysis of average days for onset of OM.

Two studies (19, 20) reported the data about average days of the onset of OM. The pooled result revealed that oral glutamine could have an effect on the onset of OM (MD, 4.11; 95% CIs, 3.49–4.73; *P* <0.01; I^2^ = 0%, seen as Figure 5). But there was a study (22) describing the onset of OM with average weeks which cannot be effectively translated into days, and another two studies (21, 24) didn't provide enough data. Hence, a qualitative analysis was performed. Tsujimoto et al. (22) found no significant difference between the two groups about the mean time of the onset of OM [Glutamine group, MD ± standard deviation (SD): 2.3 ± 0.8 weeks; Control group, MD ± SD: 2.1 ± 0.8 weeks, *P* = 0.663]. Lopez-Vaquero et al. (24) got the similar conclusion with Tsujimoto et al. (22) (Glutamine group, MD = 28.38 days; Control group, MD, 29.91 days, *P* = 0.726). Pattanyak et al. (21) reported oral glutamine was effective on delaying the onset of OM but couldn't display the specific data here.

### Severity of Oral Pain

**Figure 6:**
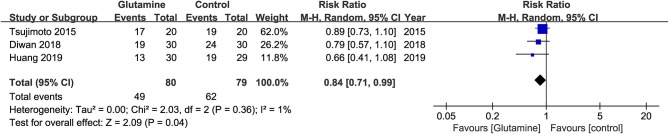
Meta-analysis of incidence of opiod use.

Three studies (20, 22, 23) were included in the pooled analysis showing that oral glutamine could significantly decrease the opioid use (RR, 0.84; 95% CIs, 0.71–0.99; *P* = 0.04; I^2^ = 1%, seen as Figure 6). Lopez-Vaquero et al. (24) found there was no significant difference between the two groups about the scores of pain evaluated by Visual Analog Scale (VAS) (Glutamine group, MD = 2.32; Control group, MD, 1.96, *P* = 0.574). However, Tsujimoto et al. (22) found patient-reported pain scores evaluated by Numerical Rating Scale (NRS) were significantly lower in the glutamine group than in the control group (*P* = 0.032).

### Glutamine-Related Adverse Events

Only two studies (22, 24) reported glutamine-related adverse events as outcome of interest and did not find occurrence of adverse events during treatment between the glutamine and the placebo groups. However, the other studies (19–21, 23) didn't design adverse events as anticipated outcome of interest.

### Publication Bias

In the present systematic review, the number of the studies that could be included to conduct a meta-analysis were all less than 10, and thus we did not perform publication bias test through drawing funnel plot.

## Discussion

RT with or without CT remains the mainstays to treat HNC (40). But after a period of treatment, there are some troubling problems which are mainly caused by the toxic damage from RT and CT on body tissue. As one of these adverse effects of RT, OM with high incidence, dose-limiting toxicity, bad impact on the QoL of patients draws great attention (9). Several regimes have been developed to prevent or treat OM among patients undergoing RT alone or concurrent with CT, but the efficacy and safety of these regimes have not yet been confirmed. Hence, it remains necessary to further develop potential alternatives in order to effectively prevent and treat OM induced by RT alone or concurrent with CT.

The present study is the first systematic review and meta-analysis of RCT to clarify the efficacy and safety of oral glutamine on preventing and alleviating OM induced RT alone or concurrent with CT among HNC patients. In the meta-analysis, we found oral glutamine may not be associated with decreased the incidence of OM (RR = 0.98, 95% CIs = 0.94–1.02) and alleviated the development of moderate or severe grade of OM (Moderate and severe OM: RR = 0.81, 95% CIs = 0.59–1.12; Severe OM: RR = 0.45, 95% CIs = 0.13–1.52). But oral glutamine could significantly delay the onset of OM (MD = 4.11, 95% CIs = 3.49–4.73) and reduce the opioid use (RR = 0.84, 95% CIs = 0.71–0.99). Moreover, glutamine-related adverse effects didn't show the significant difference.

The toxicity of ionizing radiation can stimulate the release of reactive oxygen species (ROS) from epithelial or vascular endothelial cells, which causes deoxyribonucleic acid (DNA) damage and then results in the death of epithelial and subepithelial cells of oral mucosa (41). Published evidence has demonstrated that glutamine can enhance DNA synthesis by activating ornithine decarboxylase. Moreover, glutamine may play an important role in glutathione synthesis, which can decrease the oxidative stress (42). Several clinical studies (43, 44) also established the effects of oral glutamine in preventing and treating cancer treatment related injuries. Chang and colleagues (43) found a beneficial effect of oral glutamine supplementation for the prevention from radiation-induced esophagitis in advanced patients with advanced non-small cell lung cancer who undergoing concurrent chemoradiotherapy. Randomized trial performed by Eda et al. (44) suggested that enteral glutamine minimizes radiation induced dermatitis in breast cancer patients. Nevertheless, the finding of our meta-analysis showed that oral glutamine did not decrease the incidence and alleviate the severity of radiation-induced OM, which was consistent with the result obtained by Vidal-Casariego et al. for chronic radiation enteritis (45). Among the six studies (19–24) included in the present systematic review, the conclusions of Huang et al. (23) and Lopez-Vaquero et al. (24) were consistent with our findings of our systematic review, the conclusions of the other four studies (19–22) were conflicting. We could notice that the patients in the control group received the same dose of maltodextrin with the glutamine group in the studies conducted by Huang et al. (23) and Lopez-Vaquero et al. (24), but nothing or negative subjects were given in the control group in the other studies. Moreover, Huang et al. (23) and Lopez-Vaquero et al. (24) rigorously performed the random allocation and blinding of participants, study personnel and outcome assessment, which could be more likely to ensure the real effect. But the other studies were lack of the specific report about random allocation and blinding. The two aspects of difference may be a contributor to the inconsistent conclusions.

At present, three published meta-analysis (25–27) summarized evidence of glutamine to prevent and alleviate OM during cancer therapy and they got the consistent conclusions that glutamine may have positive effects on OM. However, one of these meta-analysis failed to analyze separately the role of oral glutamine in patients with HNC (27). Another one (25) included the cohort study and didn't perform subgroup analysis. The last one (26) also included quasi randomized studies. Moreover, Worthington et al. performed a Cochrane review to comprehensively evaluate the effectiveness of prophylactic agents for OM in patients with cancer receiving treatment (28). In this review, authors also explored the efficacy of glutamine use for the prevention and treatment of OM resulted from cancer treatment. It is noted that, however, subgroup analysis according to administrations of glutamine was not designed. In addition, subgroup analysis according to cancer types was also not performed although these authors predetermined this special analysis. Meanwhile, three recent studies were not included in the three meta-analysis because their search was completed before 2016 (20, 23, 24). These limitations greatly impaired the robustness and reliability of the conclusions from the three meta-analysis. However, only RCT and patients with HNC cancer was included in the present study. More importantly, more recent trials were also incorporated in our study. Thus, we obtained more reliable and rigorous findings related to previous meta-analyses.

For the time of onset of OM, oral glutamine may have slight effect for delaying the onset of OM because only 4 days delay was found in the quantitative synthesis including two studies (19, 20). The result was similar with published systematic reviews. However, two eligible studies (22, 24) which were analyzed qualitatively supported no difference between oral glutamine and control regime in delaying onset of OM. So, it remains a conflicting whether oral glutamine has potential to delay the onset of OM. For the severity of pain, the meta-analysis of three studies (20, 22, 23) revealed glutamine could decrease the opioid use and Tsujimoto et al. (22) also found glutamine could decrease patient-reported pain scores evaluated by NRS. Thus oral glutamine may have the potential to relieve oral pain. For adverse events, only two studies (22, 24) with a small sample size reported no adverse events were found between the two groups. Hence, the result of OM-related adverse events must be cautiously considered.

Five studies (19, 20, 22–24) with 279 samples were included to perform a meta-analysis, it needs to be acknowledged that some limitations still remain. Firstly, we could not perform a subgroup analysis according to dose and frequency of oral glutamine, the scoring system, or the regime in the control group because the number of the included studies was not enough. Secondly, the five studies were carried out in Asian areas. Therefore, the findings of our systematic review need to be cautiously interpreted when glutamine is offered to patients with other backgrounds. Thirdly, the obtained findings in the present study were difficult to translate in all patients as the analyzed population is mainly represented by Asian patients.

## Conclusions

Based on the present evidence, oral glutamine may have no clinical benefits to prevent or reduce the incidence and severity of radiation-induced OM in HNC patients receiving RT alone or concurrent with CT. But it may have the potential to delay the onset OM and relieve the degree of oral pain. Nevertheless, we must cautiously interpret the results because the observed effect size for delay in mucositis start or reduction in opioid use is marginal and may not be of much clinical significance. However, further large-scale RCTs with more rigorous methodology are required to enhance the quality of evidence before making clinical decisions because of the presence of limitations.

## Data Availability Statement

The datasets analyzed in this article are not publicly available. Requests to access the datasets should be directed to Xu Tian, yxtx880919@hotmail.com.

## Author Contributions

TS conceived the study. XT and LZ captured and selected citations. XT designed the data extraction table. TS and LZ extracted data. Q-QW and TS performed all statistical analyses and prepared the manuscript draft. W-QC and X-EL revised the initial manuscript. L-LX, XT, and Y-PP critically edited language. All authors approved the final version of manuscript.

## Conflict of Interest

The authors declare that the research was conducted in the absence of any commercial or financial relationships that could be construed as a potential conflict of interest.
