# Supplementary material for: Oral Glutamine May Have No Clinical Benefits to Prevent Radiation-Induced Oral Mucositis in Adult Patients With Head and Neck Cancer: A Meta-Analysis of Randomized Controlled Trials
Source: Front Nutr. 2020 Apr 17;7:49. doi: 10.3389/fnut.2020.00049 (PMC7180868; doi:10.3389/fnut.2020.00049)
Supplement: Supplementary file 1 [file Data_Sheet_1.PDF]

Pubmed search algorithms

Search	Query
#1	Search "glutamine"[Mesh]
#2	Search glutamine[Text Word]
**#3**	**Search #1 OR #2**
#4	Search "Mucositis"[Mesh] OR "Stomatitis"[Mesh]
#5	Search (((((((Mucositis[Text Word]) OR Mucositides[Text Word]) OR mucosa irritation[Text Word]) OR mucosa inflammation[Text Word]) OR Stomatitis[Text Word]) OR Stomatitides[Text Word]) OR Oromucositis[Text Word]) OR Oromucositides[Text Word]
**#6**	**Search #4 OR #5**
#7	Search "Randomized Controlled Trial" [Publication Type] OR "Randomized Controlled Trials as Topic"[Mesh]
#8	Search random*[Text Word]
**#9**	**Search #7 OR #8**
#10	Search #3 AND #6 AND #9
